# Hepatic metastases from primary extremity leiomyosarcomas: Two case reports

**DOI:** 10.1097/MD.0000000000010598

**Published:** 2018-05-04

**Authors:** Naoki Mizoshiri, Toshiharu Shirai, Ryu Terauchi, Shinji Tsuchida, Yuki Mori, Yusei Katsuyama, Daichi Hayashi, Eiichi Konishi, Toshikazu Kubo

**Affiliations:** a Department of Orthopaedics, Graduate School of Medical Science, Kyoto Prefectural University of Medicine; b Department of Pathology, Kyoto Prefectural University of Medicine, Kamigyo-ku, Kyoto, Kyoto, Japan.

**Keywords:** extremity, hepatic metastases, leiomyosarcoma

## Abstract

**Introduction:**

Leiomyosarcoma is a highly malignant soft tissue sarcoma. Most leiomyosarcomas of the extremities metastasize initially to the lungs, with few metastasizing to the liver. Also, it is difficult to diagnose metastases to other regions of the lung during follow-up.

**Case presentation:**

The first patient was a 51-year-old Japanese woman diagnosed with a leiomyosarcoma of the left distal femur. She underwent chemotherapy, followed by wide tumor excision and reconstruction using frozen autograft with total knee arthroplasty. Eleven months later, a focal lesion was observed in her right liver, despite the absence of lung metastases. Partial hepatic resection was performed, and the hepatic lesion was diagnosed a metastasis of leiomyosarcoma. Two years later, there has been no evidence of local recurrence. The second patient was a 60-year-old Japanese male diagnosed with a leiomyosarcoma of the left thigh. He underwent preoperative chemotherapy followed by wide excision. Three years later, a focal lesion was found in his medial liver, despite the absence of lung metastases. Partial hepatic resection was performed, and the hepatic lesion was diagnosed as a metastasis of leiomyosarcoma. At the latest follow-up, there has been no evidence of local recurrence.

**Conclusions:**

The lung is the most common site of metastases from leiomyosarcomas of the extremeties, because these metastases are hematogenous. Both our patients presented with metastases of the liver, despite the absence of lung metastases. Hepatic metastasis is commonly found in computed tomography (CT) scan. Periodic CT scans of the chest and abdomen are necessary in following-up patients who undergo resection of primary leiomyosarcomas of the extremities.

## 1 Introduction

Leiomyosarcoma is a type of malignant soft tissue sarcoma of smooth muscle that may occur anywhere in the body. Leiomyosarcomas are relatively rare, accounting for approximately 10% of all soft tissue sarcoma, do not differ by sex and appear most commonly at around 65 years of age. The most common primary sites are the retroperitoneum, including intra-abdominal sites (35%); the uterus (30%); the extremities (19%); and the trunk, including the head and neck as well as the chest (16%).^[1–3]^ Wide resection is the standard treatment for leiomyosarcomas of the extremities and trunk. There is no evidence for the efficacy of neo-adjuvant and adjuvant chemotherapy.^[4]^ Patients with leiomyosarcomas of the extremities and trunk have a reported metastasis rate of 34% and 5-year and 10-year overall survival rates of 64% and 46%, respectively.^[2]^

The lungs are the most common site of metastasis from soft tissue sarcomas of the extremities and trunk. Periodic routine examination by x-rays and computed tomography (CT) of the chest has been recommended for the diagnosis of asymptomatic pulmonary metastases.^[5–7]^ Early diagnosis of lung metastases from sarcomas in other regions of the body is difficult. Although visceral and retroperitoneal soft tissue sarcomas often metastasize to the liver, hepatic metastases from soft tissue sarcomas of the extremities and trunk are rare. For example, one study found hepatic metastases in only one of 65 patients with primary soft tissue sarcomas of the extremities,^[8]^ and a second study reported hepatic metastases in 3 of 637 patients with primary soft tissue sarcomas of the extremities or trunk, with all 3 having primary tumors in the lower extremities.^[9]^ Because of their rarity, diagnosis and treatment of hepatic metastases of soft tissue sarcomas of the extremities and trunk has not been standardized. This report describes 2 patients with primary leiomyosarcomas in the lower extremities who, following treatment, developed hepatic metastases in the absence of pulmonary metastases.

## 2 Case presentation

### 2.1 Patient 1

**Figure 1:**
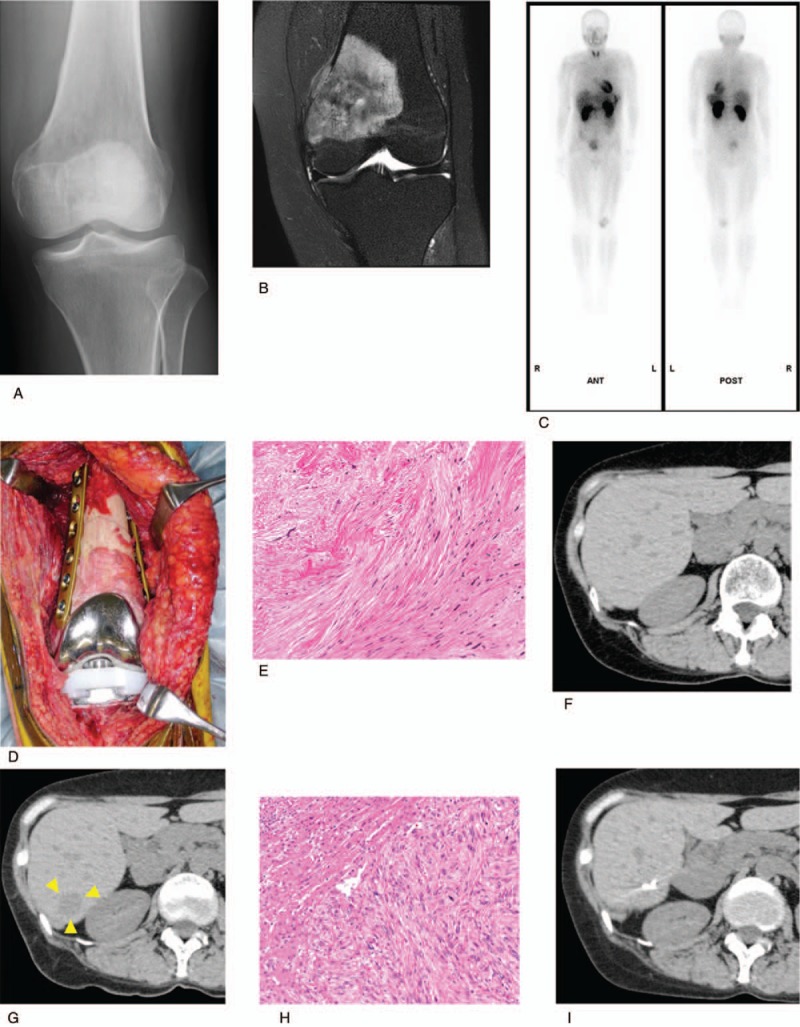
Findings in patient 1. (A) Radiograms showing osteolytic lesions and periosteal reactions of the left distal femur. (B) T2-weighted images, showing a high intensity and an internal nonuniform appearance. (C) Thallium scan, showing high accumulation in the delay phase, with no metastatic lesions. (D) Results of wide marginal excision and reconstruction using an autograft frozen in liquid nitrogen, along with total knee arthroplasty. (E) View of the resected specimen, which was pathologically diagnosed as a leiomyosarcoma. (F) CT scan 6 months after wide excision, showing the absence of focal lesion in the liver. (G) CT scan 11 months later, revealing the presence of focal lesion in the right liver (S6). (H) View of the resected liver lesion, which was pathologically diagnosed as a hepatic metastasis of leiomyosarcoma. (I) CT scan 2 years after hepatic resection, showing no evidence of local recurrence.

A 51-year-old Japanese woman had visited our hospital 4 years earlier for pain in her left knee joint. She had no abnormal findings in blood tests and physical examination of the knee showed no abnormalities. X-rays, however, showed osteolytic lesions and periosteal reactions in the left distal femur (Fig. 1A). T1-weighted magnetic resonance imaging (MRI) showed focal lesions in the distal femur and iso-signal intensity of skeletal muscle, lesions were also observed in T2-weighted, and high-intensity gadolinium-enhanced images (Fig. 1B). Thallium scans showed high accumulation during early phase and no wash out appearance in delayed phase, with no metastatic lesions (Fig. 1C). Histological examination of a CT-guided needle biopsy sample resulted in a diagnosis of leiomyosarcoma of the bone. She was treated with preoperative chemotherapy, consisting of 3 cycles of doxorubicin and cisplatin and 2 cycles of ifosfamide and etoposide. Wide excision of the tumor was followed by reconstruction using an autograft frozen in liquid nitrogen, along with total knee arthroplasty (Fig. 1D). The resected specimen was diagnosed pathologically as a leiomyosarcoma (Fig. 1E). Three weeks after surgery, she was started on postoperative chemotherapy, consisting of 2 cycles of ifosfamide and etoposide. She underwent CT scans of the chest and abdomen every 3 months. The CT scan at 6 months after tumor resection revealed no focal hepatic lesions (Fig. 1F). Eleven months later, however, a focal lesion was detected in her right liver (S6), although there were no lung metastases (Fig. 1G). A 2-deoxy-2-[fluorine-18] fluoro-D-glucose integrated with computed tomography (^18^F-FDG-PET) scan showed accumulation of ^18^F in the right liver (S6), the eleventh thoracic vertebra, and the right ilium. Histologic analysis of an ultrasound-guided needle biopsy specimen of the liver focal lesion resulted in the diagnosis of a metastatic leiomyosarcoma. A partial hepatic resection was performed to remove this lesion (Fig. 1H). Two years later, there was no evidence of local recurrence (Fig. 1I). She was administered chemotherapy, consisting of gemcitabine and docetaxel, to treat the lesions in the eleventh thoracic vertebra and right ilium.

### 2.2 Patient 2

**Figure 2:**
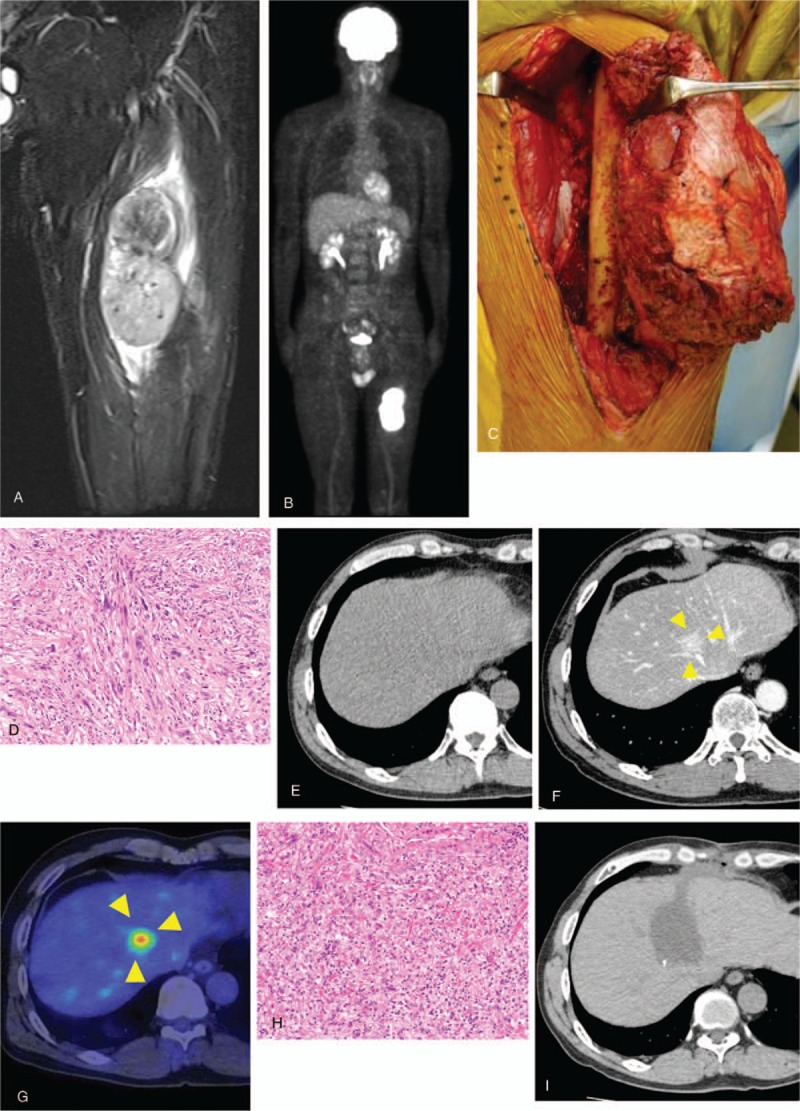
Findings in patient 2. (A) MRI showing a tumor in the left quadriceps femoris muscle, with iso-signal intensity of skeletal muscle on T1-weighted images. (B) FDG-PET scan, showing high accumulation of radioactivity in the tumor of the left thigh and no metastatic lesion. (C) Wide marginal excision of the left thigh. (D) View of the resected specimen, which was pathologically diagnosed as a leiomyosarcoma. (E) CT scan 6 months after wide excision, showing the absence of focal lesion in the liver. (F) Contrast CT scan 3 years later, revealing the presence of focal lesion in the medial liver between the S4 and S8 regions. (G) FDG-PET scan, showing accumulation of radioactivity in the medial liver. (H) View of the resected liver lesion, which was pathologically diagnosed as a hepatic metastasis of leiomyosarcoma. (I) CT scan 1 year after hepatic resection, showing no evidence of local recurrence. CT = computed tomography, MRI = magnetic resonance imaging.

A 60-year-old Japanese man had been referred to our hospital at age 55 years for a mass in his left thigh. Blood tests showed no abnormalities, and his personal and family histories were not contributory. Palpation detected an elastic, hard spherical tumor, measuring 10×5 cm, and his mobility was impaired. There were no other inflammatory findings. MRI localized the tumor to the left quadriceps femoris muscle, with T1-weighted images showing iso-signal intensity of skeletal muscle, and T2-weighted images showing high signal intensity (Fig. 2A). An ^18^F-FDG-PET scan showed high accumulation of radioactivity by the tumor, but no metastases (Fig. 2B). Histologic examination of a needle biopsy specimen resulted in a diagnosis of leiomyosarcoma. He was treated with preoperative chemotherapy consisting of 3 courses of doxorubicin and ifosfamide. Wide excision was performed (Fig. 2C), with the resected specimen diagnosed pathologically as a leiomyosarcoma (Fig. 2D). Three weeks later, he was started on postoperative chemotherapy, consisting of 2 cycles of doxorubicin and ifosfamide. He underwent CT scans of the chest and abdomen every 3 months, with the 6-month scan showing no focal lesions of the liver (Fig. 2E). Three years later, however, a contrast CT scan showed a focal lesion in the medial liver between S4 and S8, despite the absence of pulmonary nodules (Fig. 2F). In addition, an ^18^F-FDG-PET scan showed accumulation only in the medial liver (Fig. 2G). He underwent partial hepatic resection for the hepatic focal lesion, which was diagnosed histologically as a metastasis of leiomyosarcoma (Fig. 2H). At his last follow-up, there was no evidence of local recurrence (Fig. 2I).

## 3 Discussion

Leiomyosarcoma is a rare tumor, occurring most frequently in retroperitoneum, uterus, extremities, and trunk. Metastases have been reported in 34% of patients with leiomyosarcomas of the extremities and trunk leiomyosarcoma, with 61% and 87% of metastases occurring within 1 and 3 years, respectively, of diagnosis.^[2]^ The lung is the common site of metastases from leiomyosarcomas of the extremities and trunk, with metastases to the liver being rare. In contrast, retroperitoneal leiomyosarcoma can metastasize to the lung and liver. Pulmonary metastases of primary leiomyosarcomas of the extremities and trunk are indicators of hematogenous dissemination because 100% of venous return passes through the intricate capillary networks of the pulmonary vasculature.^[10–12]^ Therefore, metastases of leiomyosarcomas of the extremities and trunk must pass through the lungs, whereas no anatomical vessels pass directly through the liver. First sites of metastases of these tumors are the lungs in 88% of patients, the lymph nodes in 6%, and other soft tissue sites in 6%.^[1]^ There have been few reports of metastasis to the liver. Both our patients had leiomyosarcomas originating in the lower extremities, with metastasis to the liver but not to lungs. This condition is extremely rare and medically very interesting.

CT is usually used to detect hepatic metastases of soft tissue sarcoma. Angiography is also useful, as these lesions are hypervascular. Most hepatic metastases of soft tissue sarcoma are multiple (89%) and bilobar (74%). About 50% of patients develop hepatic metastases within 1 year of diagnosis of soft tissue sarcoma.^[9]^ However, all of these patients had intra-abdominal soft-tissue sarcomas.

Simple CT showed a clear tumorous lesion in the right liver (S6) of Patient 1 at 11 months after primary tumor resection. The metastatic lesion was solitary and resectable. Contrast CT showed a tumorous lesion in the middle liver of Patient 2 three years after primary tumor resection. This metastatic lesion was also solitary and resectable. Hepatic metastases differ in patients with primary leiomyosarcoma of the extremities and primary intra-abdominal leiomyosarcoma, in that the former are more likely to be solitary, and may take a longer time to develop following resection of the primary tumor.

In the absence of treatment, the median overall survival of patients with leiomyosarcoma and hepatic metastasis is approximately 14 months.^[13]^ These tumors have response rates of 18% to doxorubicin and 44% to the combination of doxorubicin and dacarbazine.^[9]^ Surgical resection is more effective than chemotherapy or intravascular treatment in patients with leiomyosarcoma and hepatic metastasis. In the absence of surgical resection, these patients have a 5-year survival rate of only 4%. In contrast, the 5-year survival rate following resection has been reported to range from 20% to 30%.^[13,14]^

Both of our patients were candidates for surgical resection, as both had solitary metastatic lesions. Patients 1 and 2 have survived for 2 and 1 years, respectively, after surgical resection for hepatic metastasis, with neither showing evidence of recurrence or a new metastatic lesion. Patients who have undergone surgical resection for a solitary hepatic metastasis, especially when detected at an early stage, have a good prognosis. Patients who have undergone resection of primary leiomyosarcoma of the extremities should undergo every three months CT scans of the chest and abdomen to detect hepatic metastases.

Because hepatic metastasis of primary leiomyosarcoma of the extremities is very rare, its features cannot be clarified. Assessments of an additional number of patients may reveal the characteristics of these lesions.

## 4 Conclusion

This report describes 2 patients with leiomyosarcomas originating in the lower extremities, accompanied by metastases to the liver without metastases to the lung. Periodic CT scans of the chest and abdomen are required to detect hepatic metastases following resection of the primary tumor. Patients with a solitary metastatic lesion to the liver, especially when detected at an early stage, have a good prognosis following metastasectomy.

## Acknowledgments

No other persons accept the authors contributed toward the article.

## Author contributions

**Conceptualization:** Toshiharu Shirai

**Data curation:** Eiichi Konishi.

**Formal analysis:** Eiichi Konishi.

**Investigation:** Naoki Mizoshiri, Toshiharu Shirai, Ryu Terauchi, Shinji Tsuchida, Yuki Mori, Yusei Katsuyama, Daichi Hayashi.

**Methodology:** Naoki Mizoshiri, Toshiharu Shirai, Ryu Terauchi, Shinji Tsuchida, Yuki Mori, Yusei Katsuyama, Daichi Hayashi.

**Project administration:** Toshiharu Shirai.

**Supervision:** Toshiharu Shirai, Ryu Terauchi, Eiichi Konishi, Toshikazu Kubo.

**Validation:** Toshiharu Shirai, Eiichi Konishi.

**Visualization:** Eiichi Konishi.

**Writing – original draft:** Naoki Mizoshiri.

**Writing – review & editing:** Toshiharu Shirai.

## References

[1] DeMatteo RP Shah A Fong Y Results of hepatic resection for sarcoma metastatic to liver. Ann Surg 2001;234:540–7. discussion 547-548. 11573047 10.1097/00000658-200110000-00013 PMC1422077

[2] Lang H Nussbaum KT Kaudel P Hepatic metastases from leiomyosarcoma: a single-center experience with 34 liver resections during a 15-year period. Ann Surg 2000;231:500–5. 10749609 10.1097/00000658-200004000-00007 PMC1421024

[3] Dandapani M Seagle BL Abdullah A Metastatic uterine leiomyosarcoma involving bilateral ovarian stroma without capsular involvement implies a local route of hematogenous dissemination. Case Rep Obstet Gynecol 2015;2015:950373. 26090246 10.1155/2015/950373 PMC4458360

[4] Voulalas G Giannakakis S Maltezos C Acute ischemia of extremity as a first manifestation of peripheral artery leiomyosarcoma: report of a case and review of the literature. Ann Vasc Surg 2016;34:268.e9–12. 10.1016/j.avsg.2015.11.027 26923155

[5] Gil-Sales J Vicente S Martinez N Leiomyosarcoma of the deep femoral vein. A rare cause of venous obstruction in lower limbs and an alternative diagnosis to chronic venous thrombus. Ann Vasc Surg 2012;26:1013.e1–4. 10.1016/j.avsg.2012.02.015 22944578

[6] Jaques DP Coit DG Casper ES Hepatic metastases from soft-tissue sarcoma. Ann Surg 1995;221:392–7. 7726675 10.1097/00000658-199504000-00010 PMC1234589

[7] Potter DA Kinsella T Glatstein E High-grade soft tissue sarcomas of the extremities. Cancer 1986;58:190–205. 3518911 10.1002/1097-0142(19860701)58:1<190::aid-cncr2820580133>3.0.co;2-5

[8] Gronchi A Lo Vullo S Colombo C Extremity soft tissue sarcoma in a series of patients treated at a single institution: local control directly impacts survival. Ann Surg 2010;251:506–11. 20130465 10.1097/SLA.0b013e3181cf87fa

[9] Brennan MF Antonescu CR Moraco N Lessons learned from the study of 10,000 patients with soft tissue sarcoma. Ann Surg 2014;260:416–21. discussion 421-412. 25115417 10.1097/SLA.0000000000000869 PMC4170654

[10] Smith HG Memos N Thomas JM Patterns of disease relapse in primary extremity soft-tissue sarcoma. Br J Surg 2016;103:1487–96. 27503444 10.1002/bjs.10227

[11] Grobmyer SR Maki RG Demetri GD Neo-adjuvant chemotherapy for primary high-grade extremity soft tissue sarcoma. Ann Oncol 2004;15:1667–72. 15520069 10.1093/annonc/mdh431

[12] Worhunsky DJ Gupta M Gholami S Leiomyosarcoma: one disease or distinct biologic entities based on site of origin? J Surg Oncol 2015;111:808–12. 25920434 10.1002/jso.23904

[13] Svarvar C Bohling T Berlin O Clinical course of nonvisceral soft tissue leiomyosarcoma in 225 patients from the Scandinavian Sarcoma Group. Cancer 2007;109:282–91. 17154171 10.1002/cncr.22395

[14] Gustafson P Willen H Baldetorp B Soft tissue leiomyosarcoma. A population-based epidemiologic and prognostic study of 48 patients, including cellular DNA content. Cancer 1992;70:114–9. 1606532 10.1002/1097-0142(19920701)70:1<114::aid-cncr2820700119>3.0.co;2-u

